# Descriptive sensory analysis of heat‐resistant milk chocolates

**DOI:** 10.1002/fsn3.1047

**Published:** 2019-08-20

**Authors:** Carolina B. Dicolla, Janet L. Evans, Larry L. Hainly, Sheri L. Celtruda, B. Douglas Brown, Ramaswamy C. Anantheswaran

**Affiliations:** ^1^ Department of Food Science The Pennsylvania State University University Park Pennsylvania; ^2^ Technical Center The Hershey Company Hershey Pennsylvania

**Keywords:** chocolate, heat resistance, principal component analysis, sensory analysis

## Abstract

Sensory attributes of six heat‐resistant chocolates were compared with the standard chocolate using a trained sensory panel who were trained using the Sensory Spectrum method. The panel evaluated the chocolates using three tactile and ten oral attributes at 24, 29, and 38°C. The panel demonstrated consistent rating of the various samples. ANOVA showed that all of the 13 sensory attributes (Firmness to touch, Stickiness to fingers, Snap, Abrasiveness, Hardness with incisors, Fracturability, Cohesiveness of mass, Time to melt, Firmness with tongue, Adhesiveness to teeth, Number of particles, Oily mouthcoating, and Chocolate messiness) were significantly different across the samples. A higher degree of heat resistance was identified by the panelists for the low‐fat gelatin and polyol samples at 38°C. Principal component analysis revealed two principal components; the first pricipal component described the variability due to temperature, and the second principal component described the variability brought about by the various technologies.

## 1 INTRODUCTION

Milk chocolate is a suspension of sugar, cocoa solids, and milk solids in a continuous fat phase constituted by cocoa butter and milk fat. This continuous phase is responsible for the unique melting characteristic of chocolate, whereby it is solid at room temperature (20–23.5°C) and completely melted at body temperature around 37°C (Beckett, 1999). In tropical countries or during summer days in temperate climates, traditional chocolate sticks to the wrapper, melts on the fingers, and loses its shape. There is a need for good‐tasting chocolates that can withstand higher temperatures without losing its shape and sticking to the wrapper.

Heat‐resistant chocolate has been referred within the literature as shape‐sustaining chocolate (Beckett, 1995; Kempf, 1958; Kempf & Downey, 1956; Nalur & Napolitano, 2002; O'Rourke, 1959), tropicalized chocolate products (Best, Oakenfull, Maladen‐Percy, Boehm, & Kibler, 2005, 2007; Menzi & Foucart, 1987; Ogunmoyela & Birch, 1984), thermally robust chocolate (Kealey & Quan, 1992; Willcocks et al., 2002), and temperature‐tolerant chocolate (De La Harpe & Dickerson, 2012; Dhami, O'Donnell, Harris, & Tau, 2011; Silvano & Dhami, 2012). It has been defined as a chocolate that does not adhere to the wrapper at temperatures exceeding 30°C (Schubiger & Rostagno, 1965); it will maintain its shape when exposed to temperatures above 35°C (Alander, Wärnheim, & Lühti, 1996); it is not sticky to the direct touch at 40°C (Takemori, Tsurumi, & Takagi, 1992); it will remain stiff at 50°C (Giddey & Dove, 1984); and it will have a finished flavor comparable with a conventional chocolate (Davila & Finkel, 2005; Kempf, 1958; Takemori et al., 1992). All these subjective attributes make it difficult to compare differences in the heat‐resistant chocolates described in the literature and patents.

Stortz and Marangoni (2011) have provided a review of composition and processes for production of heat‐resistant chocolate. Increasing the melting point of the fat is the easiest way to improve the heat resistance of chocolate (Pease, 1985). A secondary nonfat structure resulting from adding water, monosaccharides, amorphous sugars, polyols, fiber, starch, or protein can create heat resistance in chocolate (Afoakwa, Paterson, & Fowler, 2007; Finkel, 1990; Friedman, 1921; Killian & Coupland, 2012; Kruger & Freund, 2001; Lopez, Pariein, & Datalle, 2010).

Afoakwa et al. (2007) in reviewing the textural attributes of chocolates observed that chocolate melts in the mouth as a continuous fat phase which then inverts into a continuous water phase into which the sugar particles dissolve. A chocolate that was slow to solvate required greater effort for the tongue to compress it. The coarseness of the chocolate was observed in the inverted syrup, while a smoother chocolate was perceived as creamier. The fat and cocoa particles provided a mouthcoating sensation. Voltz and Beckett (1997) noted that only a few particles greater than 30 μm made a chocolate taste gritty while a very finely ground chocolate was difficult to swallow (Voltz & Beckett, 1997). As such, Afoakwa et al. (2007) considered the processing steps of refining, a particle size reduction step, and conching, where water was evaporated and fat and emulsifier were added, as determining many of the textural attributes of chocolate. Tempering and hardening of chocolate, where the fat was solidified into the optimum hardness, provided the remaining textural attributes of chocolate.

Sensory texture of chocolate has been extensively studied. Rodríquez, Jorge, and Beltrán (2000) observed dark, milk, and white chocolates differed by fragility, hardness, and melting. Andrea‐Nightingale, Lee, and Engeseth (2009) described dark and milk chocolates with sensory‐texture terms of hardness, cohesiveness, toothpacking, chewiness, fatty mouthcoating, toothpacking, and melting. Cagindi and Otles (2007) differentiated dark, milk, and white chocolates held at 20 and 30°C up to 12 weeks based on sensory‐texture evaluations at 20°C. Guinard and Mazzucchelli (1999) differentiated milk chocolates with varying levels of sugar and fat using sensory‐texture attributes of fatty/oily, gritty, hard, melting rate, mouthcoating, vanishing, and viscous. Haedelt, Beckett, and Niranjan (2007) differentiated aerated milk chocolates with texture attributes of hardness and melting time. Liang and Hartel (2004) showed that milk chocolates made with different types of milk powders (spray‐dried, roller‐dried, and fluidized bed) differed in “rate of meltdown while chewing,” “textural smoothness upon melting,” and “over all mouth coating sensation.” Voltz and Beckett (1997) observed that dark chocolate, when stored at either 24 or 28°C at 70% humidity, differed in first‐bite hardness and crumbliness. Medeiros de Melo, Bolini, and Efraim , Bolini and Efraim (2009) characterized milk chocolates sweetened artificially and differing in fat content with sensory attributes of sandiness, adhesion, melting rate, and hardness. Lanza, Mazzaglia, and Pagliarini (2011) differentiated specialty Sicilian chocolates with sensory attributes of firm, cohesive, adhesive, and gritty.

Sensory evaluation of heat‐resistant chocolates has been reported, but to a much lesser extent. The majority of the patents describe an informal sensory evaluation of the heat‐resistant chocolate. Some heat‐resistant chocolates were reported to have a coarser or rougher texture (Giddey & Dove, 1984; Schubiger & Rostagno, 1965), especially in those with added water (Davila & Finkel, 2005). Giddey and Dove (1984) developed a chocolate that remained stiff at 50°C yet would melt in the mouth. Ogunwolu and Jayeola (2006) reported that sensory smoothness at room temperature of heat‐resistant chocolates made with gelatin was less smooth than non‐heat‐resistant control chocolate, while a 10% starch heat‐resistant chocolate was equally smooth to the control chocolate. Subramaniam, Burke, Kristott, Groves, and Jones (1994) characterized heat‐resistant milk chocolates at 20°C with sensory hardness, meltdown time, and characteristic smoothness. These samples were differentiated by hardness and Snap after 24 hr at 20, 25, 30, 35, 40, and 50°C. Warm heat‐resistant chocolates have been compared to control chocolates at the same temperature by evaluating the chocolate's stickiness (De La Harpe & Dickerson, 2012; Marangoni, 2012) and messiness when handled (Wang & Hickey, 2012).

There are no standardized methods or protocols for sensory evaluation of heat‐resistant chocolate. However, a number of different descriptive sensory methods have been developed for other food products that could have applications for characterizing heat‐resistant chocolate. Each descriptive method identifies and calibrates attributes differently (Murray, Delahunty, & Baxter, 2001). In Flavour Profile Method and Profile Attributes Analysis (FPM/PAA), a small panel selects, defines, and rates attributes using panel‐identified standards focusing on the flavors of the foods to be rated. In Texture Profile Method (TPM), a list of texture attributes with predefined ratings anchored with preidentified standards is used to train the panelists. In Quantitative Descriptive Analysis (QDA^®^), panelists use common‐language attributes largely without anchoring to standard foods (except to resolve disagreements). In quantitative flavor profiling technique (QFPT), the panelists use the lexicon of flavorists wherein the scale is anchored extensively with standards. In the Sensory Spectrum method (SM), the panelists select appropriate attributes whose range is anchored by standard foods. In free choice profiling (FCP), panelists use their own attributes and as many as they wish and without any response‐calibrating standards.

Training in these descriptive sensory methods also differs (Murray et al., 2001). In FPM/PAA, a small panel of 4–6 panelists are trained approximately for 2–3 weeks. In TPM, at least 10 panelists are trained for as many as 130 hr over a 6‐ to 7‐month period. In QDA, panelists are trained for 10–15 hr. In QFPT, the panelists are highly trained flavorists. In SM, the panelist brings a basic understanding of the physiology and psychology of sensory perception. Extensive training as follows: 15–20 hr for terminology development, 10–20 hr scaling introduction, 15–40 hr practice, 10–15 hr focusing on small differences, and 15–40 hr arriving at a calibration. In FCP, the panelists are consumers of the particular type of product to be tested and are not trained.

The results from FPM/PAA are highly discriminating but use technical language that requires interpretation to a wider audience (Murray et al., 2001). The TPM describes food throughout its oral mastication, but the food standards to which it is anchored may not exist in other cultures or may disappear or change over time. In QDA, the differences in panelists’ ratings are used to discriminate between samples, but such differences are difficult to compare with other panels or may drift over time. Presuming one has a pool of flavorists to draw from, the description provided by QFPT is considered free of erroneous terms, while requiring interpretation for a wider audience. The results from SM are considered absolute allowing comparison with instrumental and other panels. The lack of standards in other cultures or their change over time poses challenges in such comparisons. The results of FCP only provide a rough differentiation of products, but may uncover unexpected, discriminating attributes.

Characterizing heat‐resistant chocolate requires multiple panel sessions at different temperatures. The Sensory Spectrum method will provide the consistency necessary to compare data from multiple panel sessions and hence is well suited to describe sensory attributes of heat‐resistant chocolate. The overall goal of this research was to identify sensory attributes for describing heat‐resistant milk chocolates.

## 2 MATERIALS AND METHODS

Standard milk chocolate and six heat‐resistant milk chocolates were evaluated in this study (Table 1).

**Table 1:** Formulas of milk chocolates

Ingredients (abbreviation)	Standard (S)	CSS (C)	Emulsion (E)	Low‐fat gelatin (L)	Regular‐ fat gelatin (R)	Polyol (P)	Starch (H)
Fat content (calculated) (%)	32.20	30.75	32.62	22.65	30.61	30.91	28.98
Cocoa butter (%)	19.00	18.15	17.96	12.14	18.06	18.24	17.10
Cocoa butter (emulsion) (%)			2.19				
Cocoa liquor (%)	15.15	14.47	14.32	12.91	14.40	14.54	13.64
NFDM (%)	15.00	14.33	14.18	17.37	14.26	14.40	13.50
AMF (%)	4.50	4.30	4.25	2.61	4.28	4.32	4.05
Sucrose (%)	45.93	43.86	43.40	48.75	43.66	44.09	41.33
Corn syrup (DE20) (%)		4.50					
Glycerin (%)						4.00	
Corn starch (%)							10.00
Sorbitol (30% H_2_O) (%)			3.14				
Instant Gel Schoko^®^ (%)				4.94	4.94		
Lecithin (%)	0.40	0.38	0.38	0.79	0.38	0.38	0.36
PGPR (%)			0.17	0.49			
Vanillin (%)	0.03	0.02	0.02	0.02	0.02	0.02	0.02
Total (%)	100.00	100.00	100.00	100.00	100.00	100.00	100.00

### 2.1 Ingredients

The ingredients used for making these chocolate samples were as follows: pure cane extra‐fine granulated sugar (Domino), pasteurized and spray‐dried nonfat dry milk (Darigold Inc.), Ivory Coast cocoa liquor (Blommer Chocolate Co.), glycerin 99.5% (Ruger Chemical Co.), 100% pure corn starch (Argo), sorbitol solution (70%) (Ruger Chemical Co.), Instant Gel Schoko^®^ (Gelita USA Inc.), cocoa butter (The Hershey Company), anhydrous milk fat (Dairy Farmers of America), 20 DE corn syrup solids (CSS; Grain Processing Corporation), lecithin (ADM), PGPR (Danisco), and vanillin (Citrus and Allied Essences, Ltd.).

### 2.2 Preparation of chocolates

#### 2.2.1 Standard milk chocolates (S)

A standard milk chocolate was produced as a reference based on formulas from Beckett (1999) and Stauffer (2000). The standard milk chocolate was prepared by following the steps of mixing, refining, conching, standardizing, tempering, molding, hardening, and demolding (Dicolla, 2009). The chocolate was refined in a 3‐roll refiner (29.5‐cm‐width rolls, Bühler AG) to a particle size of approximately 30 μm. The conched chocolate was standardized to bring the fat content to the specifications of the sample formulation. The standardized chocolate was tempered using the seed method. The tempered chocolate mass was molded into 10‐g squares, cooled, and demolded. During refining, the particle size was determined using a micrometer (Mitutoyo IP 65). The refined flake was dispersed in mineral oil at approximately 1:1 v/v and a drop squeezed between the anvil surfaces of the micrometer. The particle size was the extent the micrometer could be closed.

#### 2.2.2 Milk chocolate with corn syrup solids (C)

Corn syrup solids milk chocolate was reproduced based on the U.S. Patent 2904438 (O'Rourke, 1959). CSS (20 DE) were added to chocolate before refining, and the subsequent processing steps were similar to the standard milk chocolate. After the chocolate was demolded, it was exposed to 80% relative humidity at 28°C for 24 hr and then put in storage.

#### 2.2.3 Milk chocolate with added emulsion (E)

This formulation was based on European Patent 1673977 A1 (Simbürger, 2006). A water‐in‐oil emulsion was prepared by mixing cocoa butter and polyglycerol polyricinoleate (PGPR) in an emulsion mixer Silverson L4RT (Silverson Machines, Ltd.) at maximum speed, while the sorbitol solution was added slowly into it. This emulsion (5.5%) was mixed with the standard milk chocolate (94.5%) at 30°C for 20 min. The emulsion chocolate was tempered and molded in the same fashion as the standard milk chocolate and then put into storage.

#### 2.2.4 Milk chocolate with low fat and gelatin (L)

This chocolate sample was adapted from a light chocolate sample according to US Patent Application 2009/0311409 (Luccas, Efraim, & Vissotto, 2009). This formula used a hydrolyzed collagen ingredient, Instant Gel Schoko^®^, in order to replace cocoa butter without affecting the sensorial characteristics of the chocolate. All ingredients were mixed and refined together except for the milk fat and emulsifiers, which were added one hour before the end of conching. The chocolate was then tempered and molded in the same fashion as the standard milk chocolate and then put into storage.

#### 2.2.5 Milk chocolate with regular fat and gelatin (R)

The sample of regular‐fat gelatin is formulated with 4.94% Instant Gel Schoko^®^ but with a finished fat content similar to the standard milk chocolate. The same mixing, refining, conching, tempering, and molding procedures used for low‐fat gelatin were applied to the regular‐fat gelatin.

#### 2.2.6 Milk chocolate with polyol

Polyol sample was made based on U.S. Patent 6841186 (Davila & Finkel, 2005). Glycerin was added to the standard chocolate after it was standardized. The mass was mixed for an additional 20 min in order to reduce the viscosity. The polyol milk chocolate was then tempered and molded.

#### 2.2.7 Milk chocolate with starch (H)

Starch chocolate was made based on Ogunwolu and Jayeola (2006), where regular corn starch was added before the refining of ingredients. The subsequent steps were performed in the same fashion as the standard milk chocolate sample.

### 2.3 Storage conditions

All of the chocolate types including the standard milk chocolate were stored for 2–3 months, unwrapped at 21°C and 40%–60% relative humidity prior to sensory evaluation. Three batches of polyol chocolate were prepared and used as the reference in the sensory study.

### 2.4 Sensory analysis

The panel was composed of seven professionally trained panelists from The Hershey Company Technical Center (TC). The panelists had between 2 and 10 years of experience using the SM as a TC sensory panelist. Their training was conducted for 4 hr per day and 5 days per week in each of the six weeks using the Sensory Spectrum method (SM; Meilgaard, Civille, & Carr, 1991). The panelists were assessed monthly for discrimination, panel agreement, and replication using SenseTools software (v. 3.1.4, OP&P Product Research BV). Training session took place in a consensus‐panel sensory room maintained at 24°C, one of the evaluation temperatures used in the study.

The panelists were trained in a 4‐hr session for this study. The panel selected 13 attributes (three tactile and 10 oral) by consensus to evaluate the chocolate samples (Table 2). They also selected standards for the various attributes (Tables 3 and 4), and they selected the polyol sample as a reference sample (Table 5). Texture attributes already familiar to the panel were reviewed with standards and the reference sample, and four new attributes (Firmness to touch, Snap, Time to melt, and Chocolate messiness) were introduced by first rating the standards and then rating the reference sample. The polyol sample was identified by the panelists as the reference sample since it had intermediary values for all of the attributes. The standards were evaluated at 24°C. Whereas, the ratings for the reference samples at 24C were determined in the consensus‐panel room, the ratings of the reference samples at 29 and 38C were determined in the environmental chambers (Table 5). A ballot was created for each temperature and displayed a horizontal 15‐cm line scale for each attribute in the order listed in Table 2 with markings on each attribute line scale for the scores of standards (Tables 3 and 4) and the reference sample (Table 5; Chauvin, Parks, Ross, & Swanson, 2009).

**Table 2:** Sensory attributes selected by the sensory panel for characterizing heat‐resistant milk chocolate

Sensory attributes	Definition
Tactile	
Firmness to touch	The degree to which the product deforms when pressing down with the index finger.
Stickiness to fingers	The degree to which the surface of the sample adheres to the fingers when being lightly touched.
Snap	The amount of force it takes to break the product in half with the fingers.
Oral	
Abrasiveness	Degree to which the sample feels scratchy when rubbed with equal pressure on tip of tongue.
Hardness with incisors	Measure the amount of force required to bite completely through the sample with incisors.
Fracturability	The force with which a material crumbles, cracks, or shatters when placing the sample between molars and biting completely down at a fast rate.
Adhesiveness to teeth	Force required to remove material that sticks to the teeth after expectorating.
Time to melt	The time it takes the chocolate to begin to melt when massaged with the tongue.
Cohesiveness of mass	The degree to which a chewed sample holds together in a mass when chewing sample five times with molar on one side of mouth and moving sample to tongue.
Firmness with tongue	The amount of force required to compress a semisolid sample, placed between the tongue and palate with a flat tongue.
Number of particles	Number of particles perceived by tongue when mass is gently manipulated between tongue and palate.
Oily mouthcoating	The amount of oily residue felt by the tongue when moved over the surfaces of the mouth after expectorating.
Chocolate messiness	While handling and eating, measure how messy this sample is to consume.

**Table 3:** Ratings of standards used by the sensory panel for each tactile attribute

Attribute	Standard	Rating
Firmness to touch	Ready to eat pudding	0.0
Stickiness to fingers	Marshmallow	0.3
	Caramel candy chew	3.5
	Licorice candy A	6.4
	Gummy candy	11.5
	Marshmallow cut in half	13.3
Snap	Cheese sauce	0.0
	Oatmeal cream cookie	2.5
	Chocolate‐coated wafer	5.5
	Dark chocolate	10.0
	Milk chocolate C	12.8

**Table 4:** Ratings of standards used by the sensory panel for each oral attribute

Attribute	Standard	Rating
Abrasiveness	Milk chocolate A	5.0
	Potato chips	9.5
Hardness with incisors	Egg white (hard cooked)	3.4
	Hot dog (100% all beef uncooked)	4.9
	Milk chocolate A	6.7
	Dark chocolate	11.8
Fracturability	Corn muffin	0.8
	Milk chocolate B	2.3
	Crackers	4.0
	Peanuts	5.5
Cohesiveness of mass	Licorice candy B	2.1
	Hot dog (100% all beef uncooked)	4.3
	Milk chocolate A	7.0
	Brownies	9.6
Time to melt	Whipped butter	0.6
	Milk chocolate with soft center	2.8
	Milk chocolate A	4.5
	Dark chocolate	6.2
Firmness with tongue	Cheese sauce	1.0
	Peanut butter	2.3
	Chocolate‐coated fondant candy	5.4
	Brownies	11.5
	Milk chocolate C	15.0
Adhesiveness to teeth	Peanut butter	3.4
	Licorice candy A	7.6
Number of particles	Milk chocolate D	0.4
	Milk chocolate B	1.5
	Dark chocolate	3.1
	Semisweet chocolate chips	4.9
	Milk chocolate A	6.0
	Chocolate‐coated fondant candy	10.5
Oily mouthcoating		NS^a^
Chocolate messiness		NS^a^

a NS ‐ Rating determined without standards.

**Table 5:** Ratings of the reference sample of polyol milk chocolate, at 24, 29, and 38°C used by the sensory panel

Attribute	Ratings		
	24°C	29°C	38°C
Firmness to touch	13.0	10.4	4.2
Stickiness to fingers	0.8	5.8	10.6
Snap	12.7	4.7	1.9
Abrasiveness	4.1	4.7	6.0
Hardness with incisors	12.1	5.2	2.7
Fracturability	3.7	1.7	0.7
Cohesiveness of mass	6.9	9.1	11.7
Time to melt	5.9	3.7	1.8
Firmness with tongue	15.0	12.5	2.1
Adhesiveness to teeth	3.3	4.6	5.0
Number of particles	2.7	3.0	2.3
Oily mouthcoating	7.5	8.4	9.1
Chocolate messiness	3.0	5.2	11.1

The panelists performed the evaluations inside a 7‐square‐meter floor walk‐in environmental chamber (Environmental Growth Chambers) with two 8‐foot fluorescent 96‐watt tubes centered inside the chamber aligned with its length providing over‐the‐back lighting when a panelist evaluated samples. Environmental chambers were controlled at three conditions: 24°C and 50% relative humidity (RH), 29°C and 30% RH, and 38°C and 30% RH. The first condition at 24°C and 50% RH is considered typical in‐store condition when chocolate is ideally consumed. The second condition at 29°C and 30% RH is considered the upper limit for chocolate handling although less than ideal. The third condition of 38°C and 30% RH mimics a warm‐temperate climate summer day or a tropical climate.

Evaluations of the chocolate samples were conducted in five sessions, taking approximately 3 hr each. The samples were left between 16 and 20 hr in the respective environmental chambers allowing the samples to equilibrate before analysis at each temperature. Panelist was given three squares each of three different coded chocolate samples and seven squares of the reference samples. Attributes were evaluated in the order shown in Table 2. It took approximately 5 min for each panelist to evaluate the thirteen attributes for one sample and 15 min for the three coded samples. Each chocolate sample was evaluated three times in a randomized fashion blocked by temperature and evaluated at least once at each temperature during each session by every panelist. The reference sample (polyol) was presented at random three times at all three temperature as a coded chocolate sample to assess panel consistency.

### 2.5 Statistical analysis

Statgraphics (StatPoint Inc) was used for all of the statistical analysis, and they are described below.

#### 2.5.1 Sensory data analysis

The results from the descriptive sensory panel were analyzed by a multifactor analysis of variance (ANOVA), to evaluate the main effects of sample (S), Panelist (P), replicate (R), and temperature (T), and the effects of the interactions between sample and Panelist (S × P), sample and replicate (S × R), sample and temperature (S × T), Panelist and replicate (P × R), Panelist and temperature (P × T), and replicate and temperature (R × T).

#### 2.5.2 Principal component analysis

Principal component analysis (PCA) on the sensory data was conducted. Principal components with eigenvalues greater than 1.0 were retained.

## 3 RESULTS AND DISCUSSION

The reference sample (polyol) was presented at random three times at all three temperatures as a coded chocolate sample to assess panel consistency. Table 6 describes the difference in the score for each of the attributes for the coded polyol sample, and the panel agreed upon rating for the reference sample by the panelist for each attribute at each temperature. The panel demonstrated consistent rating of the unknown polyol reference sample. The panel members were largely in agreement for the attribute Stickiness to fingers (SF) evaluated at 38°C even when they disagreed with the reference sample rating at 38°C.

**Table 6:** Differences in the score for each of the attributes for the coded polyol sample and the panel agreed upon rating for the reference sample by panelist (P) for each attribute at each temperature (T in C). Attributes are as follows: Firmness to touch (FH), Stickiness to fingers (SF), Snap (SN), Abrasiveness (AB), Hardness with incisors (HI), Fracturability (FR), Cohesiveness of mass (CH), Time to melt (TM), Firmness with tongue (FT), Adhesiveness to teeth (AT), Number of particles (NP), Oily mouthcoating (OM), and Chocolate messiness (CM)

Panelist	temperature C	FH	SF	SN	AB	Attribute			TM	FT	AD	NP	OM	CM
						HI	FR	CH						
592	24	−0.05	0.03	−0.47	0.38	−0.07	−0.09	−0.05	−0.05	0.00	−0.35	0.03	0.40	−0.02
1,275	24	−0.07	0.05	−0.20	0.10	−0.04	0.05	−0.05	0.06	0.05	−0.12	0.00	−0.07	0.00
3,627	24	0.00	−0.14	−0.05	0.00	0.18	−0.32	0.50	−0.22	0.00	0.01	0.00	0.00	0.00
4,697	24	0.05	−0.05	−0.20	−0.07	−0.27	−0.67	0.48	0.40	0.00	−0.09	−0.17	0.03	0.00
6,757	24	−0.32	−0.20	0.38	0.28	0.15	0.20	−0.52	−0.55	0.00	−0.05	−0.30	0.48	0.00
9,083	24	−0.02	0.20	0.35	0.78	−0.17	−0.30	−0.50	−0.25	0.03	0.13	−0.10	−0.22	−0.07
9,386	24	0.23	−0.13	−0.06	0.14	−0.05	−0.15	−0.16	−0.06	0.48	−0.22	−0.75	0.35	0.73
592	29	0.53	0.81	1.33	0.93	−0.27	0.21	0.00	1.10	1.01	1.36	0.18	−0.37	0.73
1,275	29	0.43	0.88	1.23	0.53	−0.32	0.08	0.18	0.73	1.00	1.28	0.13	−0.27	0.71
3,627	29	0.58	0.98	1.20	1.06	−0.05	0.28	0.15	1.05	0.93	1.01	0.30	−0.30	0.91
4,697	29	0.65	0.88	1.18	0.53	−0.10	0.10	0.05	1.23	1.01	1.23	0.10	−0.32	0.76
6,757	29	0.08	0.43	1.25	0.51	−0.39	0.18	0.51	−0.42	0.56	1.45	0.48	0.68	1.38
9,083	29	−0.25	−0.12	1.18	0.75	−0.02	−0.20	−0.30	1.66	0.91	1.25	0.78	1.43	1.75
9,386	29	0.90	0.83	1.08	0.56	−0.27	0.20	0.00	1.03	0.93	1.38	0.33	−0.32	0.83
592	38	0.48	8.90	−1.67	−0.20	−0.42	0.23	2.06	0.00	0.06	1.58	0.48	0.70	−0.45
1,275	38	0.33	7.43	−0.42	0.03	−0.35	0.15	2.85	0.50	0.03	1.48	0.65	0.48	0.00
3,627	38	0.38	7.60	−0.02	0.08	0.40	0.35	2.21	0.01	0.50	1.63	0.83	0.43	0.08
4,697	38	0.05	7.90	−0.37	0.18	−0.52	0.18	2.41	0.26	0.00	1.63	0.65	0.48	0.23
6,757	38	−0.32	7.66	−0.12	0.05	−0.09	0.48	2.13	0.63	0.01	1.63	1.20	0.93	−0.57
9,083	38	1.07	5.36	0.90	0.00	−0.11	0.15	1.72	0.70	0.46	2.12	0.69	0.87	0.18
9,386	38	0.05	7.10	0.08	0.08	−0.65	0.05	2.01	0.25	−0.07	1.46	0.98	0.30	−0.70

The ANOVA of the sensory results indicated all of the 13 attributes differentiated the various samples (Table 7). The significance of the main effect of Panelist (P) and of the interaction P × R and P × S underscores the importance for ongoing training of the panelists.

**Table 7:** ANOVA of the sensory scores

Attribute	Panelist	replicate	temperature	sample	*F*‐ratio					
	P	R	T	S	P × R	P × T	P × S	R × T	R × S	T × S
Firmness to touch	11.7‡		3,521‡	12.4‡		6.8‡				4.8‡
Stickiness to fingers	15.3‡		381‡	13.1‡		12.1‡	2.2‡			8.1‡
Snap	2.2*		5,299‡	14.9‡		2.7†				4.1‡
Abrasiveness	2.6*	3.5*	83‡	9.9‡	3.2‡					2.6†
Hardness with incisors	5.3‡		12,011‡	58.4‡	3.3‡	4.1‡				10.2‡
Fracturability	3.3‡		3,443‡	37.0‡						3.3‡
Cohesiveness of mass	17.5‡		217‡	2.3*	3.2‡	9.0‡	2.1‡	2.8*		
Time to melt	10.3‡		1,013‡	25.9‡		5.7‡	3.1‡			
Firmness with tongue			10,639‡	22.2‡		3.6‡				5.2‡
Adhesiveness to teeth	7.2‡			17.3‡			3.6‡			
Number of particles	7.7‡		208‡	17.4‡		3.0‡	3.8‡			
Oily mouthcoating	5.5‡		109‡	3.7‡	3.6‡		3.2‡			
Chocolate messiness	5.3‡		3,353‡	11.6‡		4.6‡				5.6‡

* *p* < 0.05.

^†^ *p* < 0.01.

^‡^ *p* < 0.001.

**Figure 1:**
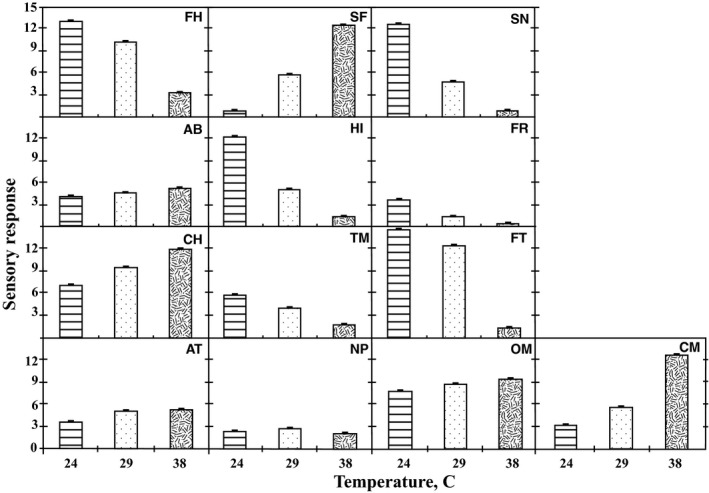
Mean value for each attribute at each temperature. Attributes are as follows: Firmness to touch (FH), Stickiness to fingers (SF), Snap (SN), Abrasiveness (AB), Hardness with incisors (HI), Fracturability (FR), Cohesiveness of mass (CH), Time to melt (TM), Firmness with tongue (FT), Adhesiveness to teeth (AT), Number of particles (NP), Oily mouthcoating (OM), and Chocolate messiness (CM)

The nature of the fat within a chocolate sample contributes substantially to the texture of the samples (Andrea‐Nightingale et al., 2009; Guinard & Mazzucchelli, 1999; Medeiros de Melo et al., 2009). The main effect of temperature (T) indicated very large *F*‐values for seven attributes (FH, SN, HI, FR, TM, FT, and CM). These seven attributes were impacted by the state of the fat in the sample and they decreased with increased temperature (Figure 1), whereas four attributes (SF, CH, OM, and CM) increased with temperatures because these attributes described the liquid fat within the sample.

**Figure 2:**
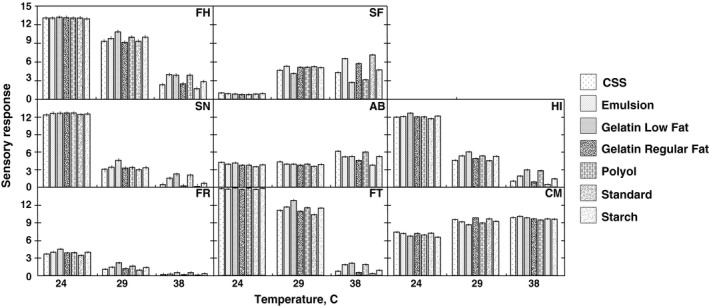
Mean value for each attribute for each sample at each temperature. Attributes are as follows: Firmness to touch (FH), Stickiness to fingers (SF), Snap (SN), Abrasiveness (AB), Hardness with incisors (HI), Fracturability (FR), Firmness with tongue (FT), and Chocolate messiness (CM); each data point is the mean of three replicate samples; vertical bars represent 95% confidence least significant difference

The magnitude of the *F* test for samples was in many cases at least an order of magnitude less than that for temperature. This implied that in general the different technologies employed to make heat‐resistant chocolate samples resulted in a smaller variation in the attributes at each temperature. Eight attributes (FH, SF, SN, AB, HI, FR, FT, and CM) were observed to have more variability at 38°C than that at the lower temperatures (Figure 2). These eight attributes showed the most differentiation at 38°C for low‐fat gelatin and polyol chocolates, suggesting these samples had the most substantial heat‐resistant structure. It has been reported that the heat‐resistant structure is a product of the nonfat phase (Stortz & Marangoni, 2011), and instrumental characterization of the melted state has been used to characterize heat‐resistant technology (Anon, 2016; Dicolla, 2009; Wang, Baker, Worthing, Gonzalez, & Mongia, 2014; Wang et al., 2015).

**Figure 3:**
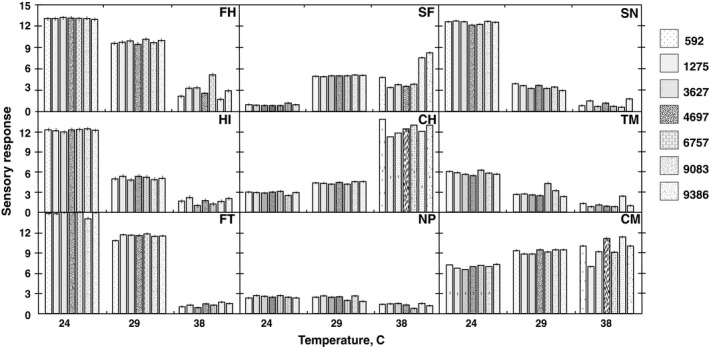
Mean value for each attribute for each panelist at each temperature. Attributes are as follows: Firmness to touch (FH), Stickiness to fingers (SF),Snap (SN), Hardness with incisors (HI), Cohesiveness of mass (CH), Time to melt (TM), Firmness with tongue (FT), Number of particles (NP), and Chocolate messiness (CM)

The interaction of P × T was significant for nine attributes (FH, SF, SN, HI, CH, TM, FT, NP, and CM), and this was also evident from the comments from the panelist that the conditions at 38°C and 30% RH for 15 min were at the limit of comfort. The most variability between panelists occurred at 38°C (Figure 3). Fang, Clausen, and Fanger (1998) explained that at higher temperatures, lower humidity would be more comfortable, as has been routinely touted in arid southern Arizona. The consistency of the replicates was indicated in the lack of significant *F*‐values for replicate or its interactions (R × T and R × S).

Given the multidimensional nature of the analysis of the data by individual attributes, PCA provided a convenient way to reduce the data to fewer orthogonal dimensions. Two principal components (PC) were identified describing 81% and 11% of the variation in the attribute scores for the samples at different temperatures (Table 8). PC1 was weighted approximately equally for 10 attributes, six positively (FH, SN, HI, FR, TM, and FT) and four negatively (SF, CH, OM, and CM). The six positively weighted attributes were observed to decrease in average response with temperature, while the four negatively weighted attributes were observed to increase in average response with temperature (Figure 1). This indicated that PC1 primarily captured the variability contributed by temperature. PC2 was primarily weighted by NP, followed by AT and then AB. NP, AT, and AB were considered properties of the nonfat portion of the samples, and thus, PC2 was considered to have captured the variability introduced by the samples and reflected the underlying heat‐resistant technology.

**Table 8:** Eigenvalues, variance, and component weights for the principal components (PC)

Attribute	PC1	PC2
Eigenvalue	10.6	1.4
Variance, %	81.4	10.9
Firmness to touch	0.299	0.123
Stickiness to fingers	−0.302	−0.069
Snap	0.299	−0.119
Abrasiveness	−0.213	0.256
Hardness with incisors	0.299	−0.100
Fracturability	0.296	−0.068
Cohesiveness of mass	−0.305	0.015
Time to melt	0.299	0.127
Firmness with tongue	0.291	0.168
Adhesiveness to teeth	−0.228	0.491
Number of particles	0.111	0.759
Oily mouthcoating	−0.297	0.052
Chocolate messiness	−0.297	−0.146

**Figure 4:**
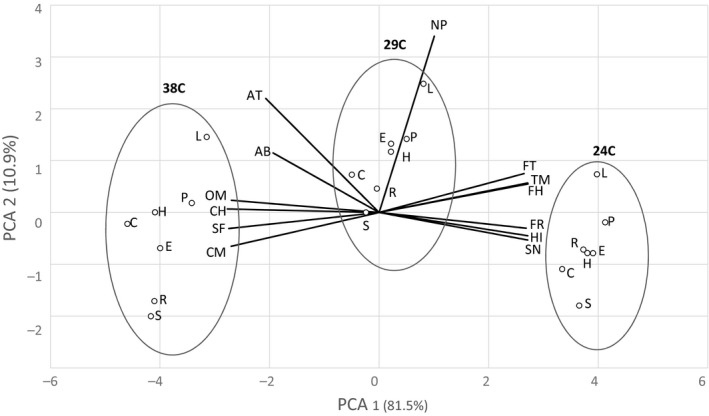
Principal component biplot for components 1 and 2. Attributes are Firmness to touch (FH), Stickiness to fingers (SF), Snap (SN), Abrasiveness (AB), Hardness with incisors (HI), Fracturability (FR), Cohesiveness of mass (CH), Time to melt (TM), Firmness with tongue (FT), Adhesiveness to teeth (AT), Number of particles (NP), Oily mouthcoating (OM), and Chocolate messiness (CM).) samples are low‐fat gelatin (L), polyol (P), starch (H), corn syrup solids (C), emulsion (E), regular‐fat gelatin (R), and standard (S)

This distribution in PC1 and PC2 space showed that samples at different temperatures were grouped together (Figure 4). Within each temperature grouping, low‐fat gelatin (L) and polyol (P) were at the top and standard (S) was at the bottom, indicating that each technology has varying impact on these attributes.

## 4 CONCLUSIONS

The overall goal of this study was to identify sensory attributes for describing heat‐resistant milk chocolates. The Sensory Spectrum method was used as a tool to characterize heat‐resistant chocolate at 24, 29, and 38°C. The panel was composed of seven professionally trained panelists between 2 and 10 years of experience using the Sensory Spectrum method. The sensory panel selected three tactile attributes for characterizing heat resistance: Firmness to touch (FH), Stickiness to fingers (SF), and Snap (SN). The ten oral attributes used were as follows: Abrasiveness (AB), Hardness with incisors (HI), Fracturability (FR), Cohesiveness of mass (CH), Time to melt TM), Firmness with tongue (FT), Adhesiveness to teeth (AT), Number of particles (NP), Oily mouthcoating (OM), and Chocolate messiness (CM). The panelists were extensively trained using various standards for anchoring the scoring of the various attributes. The addition of polyol as the reference sample provided a check on consistency of the panelists during the test, and the panel demonstrated consistent rating of the unknown polyol reference sample. The most variability between panelists occurred at 38°C evaluation. ANOVA showed that all of the 13 sensory attributes were significantly different across the samples. Seven attributes (FH, SN, HI, FR, TM, FT, and CM) were impacted by the state of the fat in the sample and they decreased with increased temperatures, whereas four attributes (SF, CH, OM, and CM) increased with temperatures because these attributes described the liquid fat within the sample. This was also reflected within the PCA. The first principal component captured the variability contributed by temperature, and the second principal component captured the variability due to the heat‐resistant structure brought about by the various technologies.

## CONFLICT OF INTEREST

The authors do not have any conflicts of interest with respect to this study.

## ETHICAL REVIEW

The sensory analysis and testing protocols were reviewed and approved by The Hershey Company Technical Center. We complied with the U.S. Federal Policy for the Protection of Human Subjects, and all of the panelists gave their informed consent prior to participation in the sensory evaluation study.
